# Dietary intervention, a promising adjunct for cancer therapy

**DOI:** 10.1038/s41392-020-00271-y

**Published:** 2020-08-24

**Authors:** Rongchen Shi, Hongming Miao

**Affiliations:** Department of Biochemistry and Molecular Biology, Third Military Medical University (Army Medical University), Chongqing, 400038 People’s Republic of China

**Keywords:** Cancer therapy, Cancer metabolism

A recent study by Irene Caffa and colleagues published in Nature demonstrated that fasting-mimicking diet (FMD) could enhance the effect of endocrine therapeutics (ET) tamoxifen and fulvestrant for breast cancer (BC) and reduce their side effects, which might provide a new adjunct to endocrine therapy for BC^1^.

**Fig. 1:**
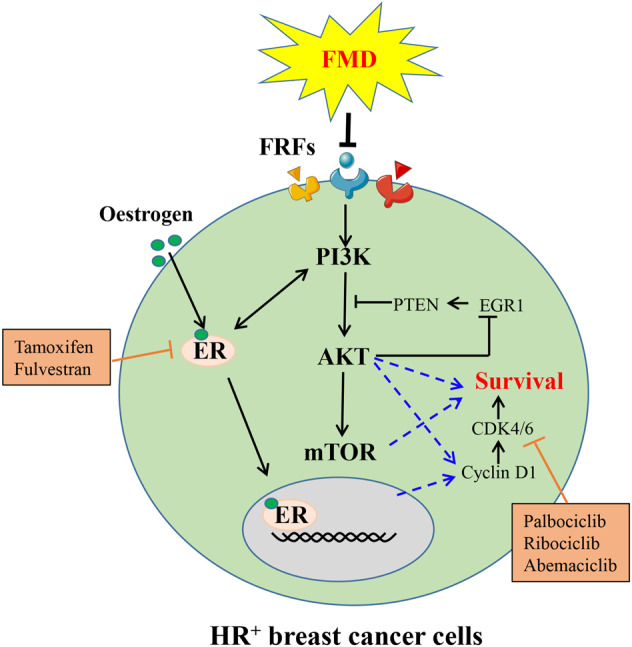
Working model for the cooperation between FMD and ET at the level of PI3K–AKT-mTOR and CCND1. FMD reduces the level of FRFs and inhibits AKT-mTOR signaling via upregulation of EGR1 and PTEN to improve the response of hormone-receptor-positive breast cancer cells to endocrine therapy. AKT inhibition also suppresses the cell cycle-related genes to cause cell cycle arrest. *FMD* fasting-mimicking diets, *FRFs* fasting-reduced factors, such as insulin, leptin, IGF1, *ER* estrogen receptor, *ET* endocrine therapy *HR*^*+*^ hormone-receptor positive

BC is a serious threat to women’s health worldwide^2^. Owing to the expression of estrogen receptor (ER) in ~75% of patients with BC and the stimulation of tumor growth by estrogen, one of the current treatment strategies for ER^+^ BC is to reduce the concentration of estrogen, also known as endocrine therapy. Unfortunately, ER^+^ BC is frequently resistant to tamoxifen and fulvestrant, which are two common endocrine therapy drugs^3^. Now, a new adjuvant therapy has been proved that FMD can promote the response of hormone-receptor-positive breast cancer to ET and enhance the antitumor effect (Fig. 1).

The activation of phosphoinositide 3-kinase (PI3K)-AKT-mammalian target of rapamycin (mTOR) axis and mitogen-activated protein kinase by growth factor signaling is one of the main factors resulting in ET resistance in BC. Meanwhile, FMD was found to reduce circulating growth factors such as insulin and IGF1. Therefore, the authors speculated that FMD might reduce the resistance of BC to ET through growth factor signaling.

The results showed that tamoxifen and fulvestrant plus FMD could lower the level of IGF1, insulin and leptin, and inhibit AKT-mTOR signaling via upregulating epidermal growth factor 1, the expression of which is associated with good prognosis in patients with BC, and PTEN, a negative regulator of AKT-mTOR signaling, and ultimately suppress tumor progression in mice.

In addition, FMD and ET also synergistically reduced the expression of cell cycle-associated genes (cyclin D1, etc) and consequently promoted cell cycle arrest in BC. Importantly, authors also found that FMD or palbociclib, a cyclin-dependent kinase 4/6 inhibitor, could postpone the occurrence of fulvestrant resistance to a similar extent. The combination of fulvestrant, FMD, and palbociclib displayed an obviously better antitumor effect.

Interestingly, the authors also found that FMD suppressed tamoxifen-induced endometrial hyperplasia. Clinical trials also showed that the prognosis of most BC patients was improved after adjuvant FMD therapy. Simultaneously, growth factor levels were reduced in almost all participants receiving FMD therapy.

Collectively, according to the mouse and clinical studies, Irene Caffa and colleagues proved that FMD could enhance the antitumor activity of ET by reducing the levels of blood growth factors, such as insulin, IGF1 and leptin, with the subsequent inhibition of the PI3K–AKT–mTOR axis.

Diet-related cancer therapy has received a lot of attention recently. Dietary habits, dietary ingredients, and dietary combinations with drugs may have unexpected effects in different cancers and in different stages of a special cancer. As described by Irene Caffa et al., FMD could directly suppress the growth of hormone-receptor-positive breast cancer cells. In fact, dietary interventions may affect all aspects of the tumor microenvironment, not just tumor cells. For example, the immune status of the tumor microenvironment is an important determinant of tumor progression. Previous studies have found that fasting and severe calorie restriction could affect the levels and function of a variety of immune cells^4,5^. During dietary restriction, the increase in the number of adipocytes and nutritive factors in bone marrow promoted the accumulation of memory T cells, thus improving the resistance to infection and tumors^5^. One of our study also found that high-fat diet treatment in an early phase could prevent peritoneal seeding of colorectal cancer by regulating innate and adaptive immunity (unpublished). Therefore, whether and how FMD would regulate antitumor immunity in BC or other cancers may be an interesting research direction in the future.

Taken together, Irene Caffa et al. suggest that FMD may have significant application potential in cancer therapy. FMD not only increases the sensitivity of BC to existing ET treatment, but also reduces their side effects. Simultaneously, this study also reconfirmed the role of diet intervention in tumor therapy, providing a theoretical basis for larger clinical studies of FMD.
